# A Retrospective Observational Study of Adverse Reactions Associated With Intravenous Immunoglobulin Infusion

**DOI:** 10.3389/fimmu.2021.740517

**Published:** 2021-09-15

**Authors:** Hidefumi Kato, Megumi Hayashi, Wataru Ohashi, Takamasa Yamaguchi, Satomi Tanaka, Ayumi Kozono, Siqiang Gao, Akiko Katai, Reiko Niwa, Tomohito Matsuo, Kazuki Ishiyama, Takanori Ando, Mika Ogawa, Takayuki Nakayama

**Affiliations:** ^1^Department of Transfusion Medicine and Cell Therapy Center, Aichi Medical University, Nagakute, Japan; ^2^Division of Biostatistics, Clinical Research Center, Aichi Medical University Hospital, Nagakute, Japan; ^3^Clinical Laboratory, Aichi Medical University, Nagakute, Japan

**Keywords:** immunoglobulin, adverse reactions, risk factors, autoimmune diseases, neuromuscular diseases

## Abstract

**Background:**

Although intravenous immunoglobulin (IVIG) therapy is generally safe and well tolerated, adverse reactions (ARs) do occur. The majority of these ARs are mild and transient. Risk factors for ARs associate with IVIG infusions are not well established. This study investigated possible risk factors influencing the occurrence of IVIG-associated ARs.

**Study Design and Methods:**

This was a retrospective observational analysis of data accumulated over 5 years, including patient demographics, clinical condition, IVIG dosing regimens, number of IVIG infusions, and any ARs.

**Results:**

ARs were associated with IVIG in 4.9% of patients and 2.5% of infusions. By univariate analyses, ARs correlated with female sex, adult age, high dose IVIG, and autoimmune disease. Multivariate logistic regression identified three statistically significant of risk factors: on a per-patient basis, being female (p=0.0018), having neuromuscular disease (p=0.0002), and receiving higher doses of IVIG per patient body weight (p<0.001), on a per-infusion basis, being female (p < 0.001), being adolescents to middle age (p < 0.001), and having neuromuscular disease (p < 0.001).

**Conclusion:**

Neuromuscular disease emerged as one of the significant factors for ARs to IVIG.

## Introduction

Intravenous immunoglobulin is widely used to treat primary and secondary immunodeficiencies, and to modulate the course of autoimmune and inflammatory conditions, such as idiopathic thrombocytopenic purpura (ITP), Kawasaki disease, eosinophilic granulomatosis with polyangiitis (EGPA), as well as various neuromuscular and dermatologic diseases (1–5). Although intravenous immunoglobulin is generally safe and well tolerated, adverse reactions (ARs) do occur. The majority of these ARs are mild and transient (6). More severe ARs, such as deep venous thrombosis, renal failure, aseptic meningitis, and hepatitis, are rarely reported. Advanced patient age, preexisting renal failure, and diabetes are associated with higher rates of IVIG infusion-related complications (7). Vascular disease and other causes of increased serum viscosity are associated with an increased risk of thromboembolism with IVIG treatment (8). However, there are limited data about ARs drawn from broad patient cohorts, so there may be risk factors not yet shown to have statistical significance. In the present study, we investigated possible risk factors influencing the occurrence of IVIG-associated ARs, with the ultimate aim of improving the comfort and safety of the patients received IVIG.

## Patients and Methods

### Study Design

We conducted a single-hospital retrospective chart review at Aichi Medical University in Japan. This study was approved by the ethics committee of Aichi Medical University, which is guided by local policy, national law, and the World Medical Association Declaration of Helsinki. We analyzed infusion protocols of all patients who received intravenous immunoglobulin (IVIG) between 1 May 2014 and 31 December 2018. Patients included in the study were diagnosed with primary immunodeficiency, hypogammaglobulinemia, antibiotic resistant sepsis, Kawasaki disease, idiopathic thrombocytopenic purpura (ITP), chronic inflammatory demyelinating polyneuropathy (CIDP), Guillain-Barre syndrome (GBS), myasthenia gravis (MG), pemphigus, polymyositis/dermatomyositis (PM/DM), eosinophilic granulomatosis with polyangiitis (EGPA), and other diseases for which IVIG is recognized as a treatment in Japan.

All patients received IVIG according to the infusion protocol of the manufacturer’s guidelines for IVIG. The course was considered to be the prescribed treatment regimen (i.e., the total dose infused over the number of infusion days) and varied between patients. IVIG was infused continuously at the rate recommended by the manufacturer’s guidelines. Infusions were started slowly (about 0.01 mL/kg body weight/minute) and were increased to the maximum prescribed rate (less than 0.06 mL/kg body weight/minute) over 1 hour.

To aggregate data from both physicians’ and nurses’ notes, prescriptions, medication administration charts, and any other documents comprising a patient’s medical record, a uniform data collection sheet was structured in accordance with the study design and objectives. Required information included: patient demographics (age, gender, and weight), indication for IVIG administration, clinical condition of patients (including vital signs), dosing regimen, the number of infusions, AR signs and symptoms, and information gained during follow-up examinations. Any reaction that occurred during an infusion and diagnosed by a physician was considered an immediate infusion-related AR. Signs and symptoms defined by the Japan Society of Transfusion Medicine and Cell Therapy (JSTMCT) are based on documents issued by the International Society of Blood Transfusion (ISBT) Working Party for Haemovigilance (9).

Reactions occurring during IVIG infusion were classified as mild, moderate, or severe. Mild reactions were those that caused only minimal discomfort and were tolerated by the subject without treatment or interruption of infusion. Moderate reactions were those that caused moderate discomfort not tolerated by the subject, for which the infusion was interrupted. Severe reactions were those that interrupted the infusion, produced sequelae, and required prolonged treatment.

### Statistical Analysis

Descriptive analyses compared baseline characteristics of patients receiving IVIG, including demographics and other potential AR risk factors, using the chi-square test for categorical variables and the Wilcoxon test for continuous variables. Crude incidence rates for ARs were estimated overall and by age, gender, disease for which IVIG was indicated and dose of IVIG. To verify any associations between the occurrence of ARs and possible risk factors (age, gender, diagnostic indication, dose of IVIG), while considering the possible influence of multiple infusions to a particular patient, logistic regressions were adjusted to consider patient characteristics [age: < 60 years *vs.* ≥ 60 years, gender: male *vs.* female, diagnostic indication: neuromuscular disease *vs.* other autoimmune diseases, dose of IVIG: < 7.0 g/kg BW *vs.* ≥ 7.0 g/kg BW (cumulative dose per patient) or < 0.45 g/kg BW *vs.* ≥ 0.45 g/kg BW (does per infusion)] as a random effect. In these regressions, the AR was the dependent variable and each factor was posited as an explanatory variable. Logistic regression was used to estimate the odds ratios (ORs) and 95% confidence intervals (CIs) of AR occurrence for each factor. In all analyses, conducted using SPSS (SPSS software package version 23.0, IBM, Tokyo, Japan), a significance threshold of <0.05 was used.

## Results

### Patient Demographics

During this study, 748 patients received IVIG (**Table 1**), of whom 389 were male and 359 patients were female. Their mean age was 41.7 (range 0-99) years. Just over half (377) had autoimmune diseases, including neuromuscular diseases (n = 71), dermatologic diseases (n = 58), ITP (n = 61), EGPA (n = 16), and Kawasaki disease (n = 171). The neuromuscular diseases included CIDP (n = 27), GBS (n = 20), and MG (n = 24). The 58 dermatologic diagnoses included pemphigus (n = 24) and PM/DM (n = 34). Other patients had hypogammaglobulinemia (n = 64), sepsis (n = 296), and various other conditions (n = 11).

**Table 1:** Patients demographics.

				Patients	Infusions
**Numbers**				748	4070
	Male			389	1658
	Female			359	2412
**Mean age (years) (range)**				41.7 (0-99)	
**Indications for receiving IVIG (n)**					
	Autoimmune			377	2856
		Neuromuscular		71	1038
			CIDP	27	495
			GBS	20	425
			MG	24	118
		Dermatologic		58	1102
			Pemphigus	24	835
			PM/DM	34	217
		ITP		61	341
		EGPA		16	153
		Kawasaki disease		171	222
	Hypogammaglobulinaemia			64	288
	Sepsis			296	904
	Others			11	22

CIDP, chronic inflammatory demyelinating polyneuropathy; GBS, Guillain-Barre syndrome; MG, myasthenia gravis; PM/DM, polymyositis/dermatomyositis; ITP, idiopathic thrombocytopenic purpura; EGPA, eosinophilic granulomatosis with polyangiitis (Churg-Strauss syndrome).

During this study, a total of 4070 infusions were administered, of which 1658 were for males and 2412 were for females. Just over half (2856) were autoimmune diseases, including neuromuscular diseases (n=1038), dermatologic diseases (n=1102), ITP (n=341), EGPA (n=153), and Kawasaki disease (n=222). Other infusions were for hypogammaglobulinemia (n=288), sepsis (n=904), and various other conditions (n=22).

Average doses of IVIG per patient body weight varied by diagnosis: 3.7 g/kg for autoimmune diseases, 0.6 g/kg for hypogammaglobulinemia, 0.4 g/kg for sepsis, and 1.2 g/kg for other conditions in aggregate. Thus, the average dose of IVIG for autoimmune diseases was highest by a substantial margin. The mean daily dose per body weight of IVIG varied by diagnosis: 0.45 g/kg for neuromuscular diseases, 0.42 g/kg for dermatologic diseases, 0.49 g/kg for ITP, 0.39 g/kg for EGPA, 1.55 g/kg for Kawasaki disease, 0.15 g/kg for hypogammaglobulinemia, 0.15 g/kg for sepsis, and 0.61 g/kg for other conditions. Thus, the daily dose of IVIG for autoimmune diseases was highest by a substantial margin. Furthermore, the mean course dose per body weight of IVIG for autoimmune diseases (1.76 g/kg) was higher than those for non-autoimmune diseases (0.25 g/kg) excepting other conditions (0.98 g/kg). Thus, patients with non-autoimmune diseases including hypogammaglobulinemia and sepsis received low-dose IVIG (less than 0.7 g/kg body weight per course). In contrast, patients with autoimmune diseases and other conditions received high-dose IVIG (more than 0.7 g/kg body weight per course).

As for the gender distribution of patients with autoimmune diseases, the male-to-female ratio for neuromuscular diseases (male/female: 1.09) was higher than that for non-neuromuscular diseases (male/female: 0.78). However, there was no significant difference in these ratios (p = 0.186) (**Table 2**). Furthermore, among neuromuscular diseases, the male-to-female ratios for CIDP, GBS, and MG were 1.25, 1.22, and 0.85, respectively (p = 0.601). In contrast, on the infusions, the male-to-female ratio for neuromuscular diseases (male/female: 0.95) was significantly higher than that for no-neuromuscular diseases (male/female: 0.33) (p < 0.001).

**Table 2:** Patients demographics on gender in autoimmune diseases.

		Patient basis (n=377)		Infusion basis (n=2856)	
		Gender ratio(male/female)	*P* value	Gender ratio(male/female)	*P* value
**Neuromuscular**		1.09	0.186*	0.95	<0.001*
	CIDP	1.25		1.35	
	GBS	1.22		1.36	
	MG	0.85	0.601^†^	0.57	<0.001^†^
**Non-neuromuscular**		0.78		0.33	
	Dermatologic	0.53		0.21	
	ITP	0.56		0.41	
	EGPA	0.45		0.53	
	Kawasaki disease	1.04		1.04	

*p values refer to gender ratio between neuromuscular and non-neuromuscular diseases.

^†^p values refer to gender ratio among neuromuscular diseases (CIDP/GBS/MG).

### Adverse Reactions

Of 360 patients received low-dose IVIG (less than 0.7 g/kg body weight per course), 3 (0.8%) experienced ARs. As shown in **Table 3**, the incidence of ARs was not different between males (0.9%) and females (1.3%). Furthermore, the incidence of ARs for patients age under 15 years was not significantly higher than for patient age 15 years and over (p = 0.245). On a per infusion basis, 5/1,192 infusions of IVIG (0.4%) administered were associated with ARs. The average doses (g) of IVIG per body weight (kg) for patients and infusions with ARs (1.4 g/kg and 0.19 g/kg, respectively) were not significantly higher than for patients and infusion without ARs (0.4 g/kg and 0.16 g/kg, respectively) (p = 0.395 and 0.564, respectively).

**Table 3:** Factor-specific incidences of adverse reactions to low-dose IVIG*.

		Patient basis (n=360)			Infusion basis (n=1192)		
		Adverse reaction			Adverse reaction		
		No	Yes	*p* value	No	Yes	*p* value
Gender, n (%)							
	Male	211 (99.1)	2 (0.9)		680 (99.6)	3 (0.4)	
	Female	145 (98.7)	2 (1.3)	1.00	507 (99.6)	2 (0.4)	1.00
Age, n (%)							
	< 15 years	69 (97.2)	2 (2.8)		181 (97.2)	3 (1.6)	
	≥ 15 years	287 (99.3)	2 (0.7)	0.245	1006 (99.3)	2 (0.2)	0.025
IVIG doses (average, g/kg BW)		0.4^†^	1.4^†^	0.395	0.16	0.19	0.564
Indication, n (%)							
	Hypogammaglobulinaemia	63 (98.4)	1 (1.6)		286 (99.4)	2 (0.6)	
	Sepsis	293 (99.0)	3 (1.0)		901 (99.7)	3 (0.3)	

*The low-dose IVIG is less than 0.7 g/kg body weight per course.

^†^The values indicate the cumulative doses from multiple treatment of IVIG on one patient.

BW, body weight of patient.

Among patients received high-dose IVIG (more than 0.7 g/kg body weight per course), ARs were recorded in 8.5% of patients and in 3.4% of infusions (**Table 4**). The incidence of ARs per patient was significantly higher for females (12.3%) *vs.* males (4.0%) (p = 0.003). The mean ages of patients with and without ARs were 52.8 and 28.4 years, respectively. Furthermore, the incidence of ARs for patients age 15 years and over was significantly higher than for patients age under 15 years (p < 0.001). Thus, among patients who received IVIG, ARs were more likely to occur in adults than in children. The average doses (g) of IVIG per body weight (kg) for patients with ARs (10.9 g/kg body weight) were significantly higher than for patients without ARs (3.0 g/kg body weight) (p = 0.003).

**Table 4:** Factor-specific incidences of adverse reactions to high-dose IVIG*.

			Patient basis (n=388)			Infusion basis (n=2888)		
			Adverse reaction			Adverse reaction		
			No	Yes	*p* value	No	Yes	*p* value
Gender, n (%)								
	Male		169 (96.0)	7 (4.0)		961 (98.6)	14 (1.4)	
	Female		186 (87.7)	26 (12.3)	0.003	1820 (95.6)	83 (4.4)	< 0.001
Age, n (%)								
	< 15 years		196 (99.0)	2 (1.0)		314 (99.4)	2 (0.6)	
	≥ 15 years		159 (83.7)	31 (16.3)	< 0.001	2467 (96.3)	95 (3.7)	0.003
IVIG doses (average, g/kg BW)			3.0^†^	10.9^†^	0.003	0.65	0.48	0.004
Indication, n (%)								
	Autoimmune		344 (91.2)	33 (8.8)		2759 (96.6)	97 (3.4)	
		Neuromuscular	52 (73.2)	19 (26.8)		973 (93.7)	65 (6.3)	
		Dermatologic	51 (87.9)	7 (12.1)		1078 (97.8)	24 (2.2)	
		ITP	59 (96.7)	2 (3.3)		339 (99.4)	2 (0.6)	
		EGPA	13 (81.2)	3 (18.8)		149 (97.4)	4 (2.6)	
		Kawasaki disease	169 (98.8)	2 (1.2)		220 (99.1)	2 (0.9)	
	Others		11 (100.0)	0 (0.0)		22 (100.0)	0 (0.0)	

*The high-dose IVIG is more than 0.7 g/kg body weight per course.

^†^The values indicate the cumulative doses from multiple treatment of IVIG on one patient.

BW, body weight of patient.

The incidence of ARs per infusion was significantly higher for females (4.4%) *vs.* males (1.4%) (p < 0.001). Similarly to the results on a per-patient basis, the incidence of ARs for patients age 15 years and over was significantly higher than for patients age under 15 years (p = 0.003). However, contrary to the results on a per-patient basis, the average doses (g) of IVIG per body weight (kg) for infusions without ARs (0.65 g/kg body weight) were significantly higher than for infusions with ARs (0.48 g/kg body weight) (p = 0.004).

Of 377 patients with autoimmune diseases, 33 (8.8%) experienced ARs. In contrast, just 1 of 64 patients (1.6%) with hypogammaglobulinemia, 3 of 296 patients (1.0%) with sepsis, and 0 of 11 patients with other diseases experienced ARs. Furthermore, of 2856 infusions with autoimmune diseases, 97 (3.4%) experienced ARs. In contrast, 2 of 288 infusions (0.7%) with hypogammaglobulinemia, 3 of 904 infusions (0.3%) with sepsis, and 0 of 22 infusions with other diseases experienced ARs. Thus, the incidence of ARs to IVIG for autoimmune diseases was higher than on non-autoimmune diseases. Overall, gender, age, IVIG dose, and indication (diagnosis) were found to be associated with AR incidence.

Therefore, logistic regression was used to identify characteristics associated with ARs in the subgroups with high incidence of ARs by univariate analyses (age ≥ 15 years and autoimmune diseases). Of 184 patients and 2548 infusions in this subgroup, 31 patients (16.8%) and 95 infusions (3.7%) experienced ARs. 19 of 67 patients (28.4%) and 65 of 1014 infusions (6.4%) with neuromuscular disease experienced ARs. In contrast, 12 of 117 patients (10.3%) and 30 of 1534 infusions (2.0%) with non-neuromuscular diseases experienced ARs. The incidences of ARs per patient and per infusion were higher for females (25/108 patients: 23.1% and 82/1714 infusions: 6.5%, respectively) *vs.* males (6/76 patients: 7.9% and 13/834 infusions: 3.8%). The incidence of ARs for patients age between 15 and 59 years (20/89 patients: 22.5%) was significantly higher than for patients age 60 years and over (11/95 patients: 11.6%). Similarly, on a per-infusion basis, the incidence of ARs for patient age between 15 and 59 years (79/1392 infusions: 5.7%) was significantly higher than for patients age 60 years and over (16/1156 patients: 1.4%). The incidence of ARs for patients received a total dose of IVIG above 7.0 g/kg bodyweight (15/29: 51.7%) was higher than that for patients received less than 7 g/kg bodyweight (16/155: 10.3%). In contrast, the incidence of ARs on less than 0.45 g/kg bodyweight per infusion (77/1961: 3.9%) was higher than that on above 0.45 g/kg bodyweight per infusion (18/587: 3.1%). As shown **Table 5**, on a per-patient basis, significant univariate risk factors for ARs to IVIG included gender (male/female) (odds ratio [OR], 0.285; 95% CI, 0.111-0.733), neuromuscular disease (OR, 3.464; 95% CI, 1.557-7.703), and dose of IVIG per body weight (OR, 9.308; 95% CI, 3.809-22.744), while age was not significant (p = 0.0521). Furthermore, multivariate logistic regression of risk factors for ARs to IVIG identified being female (p = 0.0018), having neuromuscular disease (p = 0.0002), and receiving higher doses of IVIG per body weight (p < 0.001) as significant risk factors. On the other hand, there was no correlation between neuromuscular disease and dose of IVIG per patient bodyweight as risk factors for ARs by Spearman’s rank correlation coefficient. On a per-infusion basis, significant univariate risk factors for Ars to IVIG included gender (male/female) (odds ratio [OR], 95% CI), neuromuscular disease (OR, 3.434; 95% CI, 2.211-5.333), and age (OR, 0.233; 95% CI, 0.136-0.402), while dose of IVIG per body weight per infusion was not significant (p = 0.336). Furthermore, multivariate logistic regression of risk factors for ARs to IVIG identified being female (p < 0.001), having neuromuscular disease (p < 0.001), and adolescents to middle age (age < 60 years) (p < 0.001) as significant risk factors.

**Table 5:** Logistic regression analyses of risk factors for adverse reaction to IVIG therapy.

		Univariate			Multivariate		
		Odds	95%CI	P value	Odds	95%CI	P value
Patient basis (n = 184)							
	Male/Female	0.285	0.111-0.733	0.0092	0.156	0.049-0.501	0.0018
	Age ≥ 60 years	0.452	0.203-1.007	0.0521	0.542	0.21-1.396	0.2043
	Neuromuscular disease	3.464	1.557-7.703	0.0023	6.914	2.485-19.237	0.0002
	Dose ≥ 7.0 g/kg BW*	9.308	3.809-22.744	<0.001	12.259	4.28-35.108	<0.001
							
Infusion basis (n = 2548)							
	Male/Female	0.315	0.175-0.569	<0.001	0.178	0.097-0.328	<0.001
	Age ≥ 60 years	0.233	0.136-0.402	<0.001	0.235	0.134-0.414	<0.001
	Neuromuscular disease	3.434	2.211-5.333	<0.001	4.635	2.92-7.356	<0.001
	Dose ≥ 0.45 g/kg BW	0.774	0.459-1.304	0.336	1.045	0.598-1.821	0.878

*The values indicate the cumulative doses from multiple treatment of IVIG on one patient.

BW, body weight of patient.

The most commonly documented ARs were fever (54.1%) and headache (51.4%), followed by flu-like symptoms (16.2%) and rash (10.8%) on a per-patient basis (**Table 6**). The most commonly documented ARs were fever (47.1%) and headache (42.2%), followed by flu-like symptoms (8.8%) and rash (3.9%) on a per-infusion basis. Also, the distribution of ARs among patients with autoimmune diseases was almost the same as among all patients. Among patients with neuromuscular diseases, the most commonly documented ARs were headache (per patient: 73.7%, per infusion: 49.2%) and fever (per patient: 57.9%, per infusion: 46.2%). However, among patients without neuromuscular diseases, the most documented ARs was fever (per patient: 42.9%, per infusion: 43.8%).

**Table 6:** Clinical presentations of IVIG-related adverse reactions.

ARs	All patients		Autoimmune diseases					
			All		Neuromuscular diseases		Other^#^	
	Patients n = 37	Infusions n = 102	Patients n = 33	Infusions n = 97	Patients n = 19	Infusions n = 65	Patients n = 14	Infusions n = 32
Headache	19 (51.4)	43 (42.2)	19 (57.6)	43 (44.3)	14 (73.7)	32 (49.2)	5 (35.7)	11 (34.4)
Fever	20 (54.1)	48 (47.1)	17 (51.6)	44 (45.4)	11 (57.9)	30 (46.2)	6 (42.9)	14 (43.8)
Flu-like symptoms	6 (16.2)	9 (8.8)	5 (15.2)	8 (8.2)	3 (15.8)	5 (7.7)	2 (14.3)	3 (9.4)
Hypertension	1 (2.7)	1 (1.0)	1 (3.0)	1 (1.0)			1 (7.1)	1 (3.1)
Dyspnea	1 (2.7)	1 (1.0)	1 (3.0)	1 (1.0)	1 (5.3)	1 (1.5)		
Chest pain	2 (5.4)	2 (2.0)	2 (6.1)	2 (2.1)	2 (10.5)	2 (3.1)		
Rash	4 (10.8)	4 (3.9)	3 (9.1)	3 (3.1)			3 (21.4)	3 (9.4)

ARs, adverse reactions. All table values are n (%).

^#^Other of autoimmune diseases are those without neuromuscular manifestations.

## Discussion

We retrospectively analyzed ARs to IVIG with uniform data collection forms and consistent methodology to aggregate 5 years of data from multiple departments. The incidence of ARs was 4.9% of all patients treated with IVIG, among whom univariate analyses showed AR risk factors to be female sex, adult age, higher doses of IVIG, and autoimmune disease. Furthermore, when multivariate logistic regression analyses for AR risk factors were applied to high risk subgroups, being female (p = 0.0018), having neuromuscular disease (p = 0.0002), and receiving higher doses of IVIG per patient body weight (p < 0.001) were significant risk factors. On the other hand, the incidence of ARs was 2.5% of all infusions administered IVIG, among which univariate analyses showed AR risk factors to be female sex, adult age, and autoimmune disease. Furthermore, when multivariate logistic regression analyses for AR risk factors were applied to high risk subgroups, being female (p < 0.001), having neuromuscular disease (p < 0.001), and being adolescents to middle age (p < 0.001) were significant risk factors.

IVIG has been widely used for a variety of conditions, including primary and secondary immunodeficiency diseases, autoimmune diseases (including ITP, CIDP, Guillain-Barre syndrome, PM/MD, Kawasaki disease, etc.), and sepsis. Although a large number of clinical trials have demonstrated that IVIG is generally safe and well tolerated, various ARs do occur. The reported incidence of ARs varies widely, from 1% to more than 50% of patients, depending on the study (10–14). ARs are rare among immunodeficient patients when receiving the same dose as previously well tolerated at regular intervals. Struff et al. (15) reported that among 1705 immunodeficient patients at 72 centers, given 15,548 infusions of the same product, only 10 patients (0.6%) ever had an AR, with their per-infusion rate an order of magnitude lower, at 0.064%. On the other hand, the highest rates of ARs occur among non-immunodeficient and non-septic patients with autoimmune diseases receiving high doses IVIG, e.g., 1 to 2 g/kg. Donofrio et al. (16) reported that 18% of infusions and 55% of 113 patients with chronic inflammatory polyneuropathy developed ARs to IVIG. Indeed, in this study, although the incidences of ARs among those with non-autoimmune diseases, such as hypogammaglobulinemia and sepsis, were low (1.6% and 1.0%, respectively), the incidences of ARs among those with autoimmune diseases was high (8.8%). In addition, the IVIG doses for patients with autoimmune diseases were higher than for those with non-autoimmune diseases. Furthermore, the present and previous studies (14, 17) have reported that the mean course doses per bodyweight (g/kg) for patients with ARs (1.9 g/kg) were significantly higher than for patients without ARs (1.1 g/kg) (p < 0.001) (data not shown). The mean course dose per bodyweight of IVIG for autoimmune diseases (1.76 g/kg) was higher than those for non-autoimmune diseases (0.25 g/kg) excepting other conditions (0.98 g/kg). We speculate that these different incidences of ARs between the non-autoimmune diseases cohort (including immunodeficiency, sepsis, etc.) and the autoimmune diseases cohort might be due to different IVIG infusion doses.

The previous study (18) has reported that the higher infusion rates were strongly associated with ARs. In addition, reducing the infusion rate after the occurrence of ARs may improve symptoms of ARs (19). In this study, ARs can be avoided by beginning the infusion slowly and gradually increasing the rate. In contrast, the previous study (20) reported that the infusion rate was significantly slower in patients with ARs after IVIG infusion. Thus, we speculate that the infusion rates were not associated with ARs, because the infusion rates were not significantly different among patients received IVIG due to manufacturer’s guidelines in our study.

Our data show that ARs were more likely to occur in adults (6.9%) than in children (1.5%), in contrast to a previous study that showed no such difference (19). However, the patient cohort in that previous study consisted only of those with primary immunodeficiency diseases and did not include anyone with autoimmune diseases. Our study patients included those with immunodeficiency diseases, sepsis, and autoimmune diseases. Furthermore, although the age was not reflected in the incidence of ARs in the low-dose IVIG cohort (including immunodeficiency and sepsis), ARs were more likely to occur in adults (16.3% on a per-patient basis, 3.7% on a per-infusion basis) than in children (1.0% on a per-patient basis, 0.6% on a per-infusion basis) in high-dose IVIG cohort (including autoimmune diseases) (**Table 4**). Thus, the influence of the age on ARs among those with autoimmune diseases need further investigation.

Our study quantified risk factors for the occurrence of ARs with IVIG therapy among those with a high incidence of ARs, i.e., age ≥ 15 years and autoimmune diseases. By multivariate logistic regression analyses on a per-patient basis, female gender, neuromuscular diseases, and higher doses of IVIG per patient bodyweight emerged as risk factors for ARs, while younger age (less than 60 years old) was not (**Table 5**). In contrast, on a per-infusion basis, multivariate logistic regression of risk factors for ARs to IVIG identified adolescents to middle age (age < 60 years) (p < 0.001) as significant risk factors. Waheed et al. (12) reported that multivariate analyses identified the following risk factors for ARs on a per-infusion: younger age. Thus, the age as risk factors for ARs was reflected on a per-infusion basis rather than on a per-patient basis. In particular, the likelihood of ARs to IVIG correlated with having neuromuscular diseases (odds: 6.914) among patients with any autoimmune disease. In fact, in that subgroup, the incidence of ARs for neuromuscular diseases was 28.4% (data not shown), consistent with other studies (12, 14, 21) that have reported ARs in more than 20% of patients with neuromuscular diseases. Wietek et al. (22) analyzed data from 112 patients with ITP receiving IVIG, in which there were five cases with at least one AR. In addition, ARs to IVIG were experienced by up to 10% of patients with dermatologic diseases (1). A possible reason for higher incidence of ARs with neuromuscular diseases is that the total doses of IVIG for patients with neuromuscular diseases might be higher than those with non-neuromuscular diseases. The previous study reported that ARs occurred more often during treatment in the group that received a high total dose, suggesting that this side effect was dose-dependent (14). We showed that the average total dose per patient with ARs was significantly higher than among those without ARs. Furthermore, by multivariate logistic regression, total dose per patient emerged as a risk factor for ARs (odds: 12.259). In addition, by multivariate logistic regression on a per-infusion basis, dose per infusion was not emerged as a risk factor for ARs (odds: 1.045). However, there was no correlation between neuromuscular disease and total dose of IVIG per patient in risk factors of ARs to IVIG by Spearman’s rank correlation coefficient. Indeed, there is no difference in total average dose per patient between those with neuromuscular diseases and those with dermatologic diseases (data not shown). Also, the female sex was one of AR risk factors among patients with autoimmune diseases. However, when the gender distributions were investigated in this study, neuromuscular diseases were found to be male-significant compared to non-neuromuscular diseases both on the patient basis and the infusion basis (**Table 2**). Therefore, the hypothesis that there was higher rate of ARs to IVIG among patients with neuromuscular diseases based on total dose of IVIG and gender distributions is not supported by our results. While the reason for this is unclear, this implicates neuromuscular disease as a risk factor for ARs to IVIG.

The most common immediate ARs are headache, fever, flu-like symptoms (23), chest pain, and rash. These usually are mild and occur within an hour of starting an infusion and disappear within 6 hours. Particularly, headaches are a common complaint during or shortly after IVIG infusions. Headaches are common with high dose IVIG therapy, typically used in autoimmune diseases. In this study, headaches occurred in 57.6% of patients with autoimmune diseases. In particular, most ARs in patients with neuromuscular diseases were headache (73.7%), whereas headache in patients with other autoimmune diseases were observed only 35.7%. Bertorini et al. (14) reported that the proportion of headaches among all ARs in patients with neuromuscular diseases was higher than in patients with non-neuromuscular autoimmune diseases. The mechanism of IVIG-induced headache is unclear, but patients with neuromuscular diseases frequently have headaches in conjunction with IVIG therapy.

The previous study (24) has reported that headache could be prevented by changing from IVIG treatment to subcutaneous immunoglobulin (SCIG) treatment. However, patients with frequent ARs were autoimmune diseases that treated high-dose IVIG in this study. It is a small amount when patients treated by SCIG. Therefore, it is possible that patients with autoimmune diseases will be given frequently SCIG.

We conclude that having neuromuscular disease, being female, and receiving higher doses of IVIG are all risk factors for infusion-related ARs. Related to this, we found no correlation between neuromuscular disease and the total dose of IVIG per patient in the risk of ARs. In particular, the majority of ARs in patients with neuromuscular diseases are headaches. Although having neuromuscular disease emerged as a factor contributing to ARs, a limitation of our study is that it is a retrospective analysis. Despite this limitation the study provides insight into risks for ARs in patients receiving IVIG. In the future, more elaborate analyses of data collected from individual patients may allow recommendations to emerge that improve the safety and comfort of IVIG therapy.

## Data Availability Statement

The original contributions presented in the study are included in the article/supplementary material. Further inquiries can be directed to the corresponding author.

## Ethics Statement

The studies involving human participants were reviewed and approved by the Aichi Medical University (Approval No. 2021-043). The patients/participants provided their written informed consent to participate in this study.

## Author Contributions

HK and MH: design and concept of study, data analysis, drafting and revision of manuscript. WO: data analysis, drafting and revision of manuscript. TY, ST, AKo, SG, AKa, RN, TM, KI, TA, MO, and TN: data collection, drafting and revision of manuscript. All authors contributed to the article and approved the submitted version.

## Conflict of Interest

The authors declare that the research was conducted in the absence of any commercial or financial relationships that could be construed as a potential conflict of interest.

## Publisher’s Note

All claims expressed in this article are solely those of the authors and do not necessarily represent those of their affiliated organizations, or those of the publisher, the editors and the reviewers. Any product that may be evaluated in this article, or claim that may be made by its manufacturer, is not guaranteed or endorsed by the publisher.
